# Accelerated bio‐cognitive aging in Down syndrome: State of the art and possible deceleration strategies

**DOI:** 10.1111/acel.12903

**Published:** 2019-02-15

**Authors:** Claudio Franceschi, Paolo Garagnani, Noémie Gensous, Maria Giulia Bacalini, Maria Conte, Stefano Salvioli

**Affiliations:** ^1^ IRCCS Istituto delle Scienze Neurologiche di Bologna Bologna Italy; ^2^ Lobachevsky State University of Nizhny Novgorod Nizhny Novgorod Russia; ^3^ Department of Experimental, Diagnostic and Specialty Medicine (DIMES) University of Bologna Bologna Italy; ^4^ Clinical Chemistry, Department of Laboratory Medicine Karolinska Institutet at Huddinge University Hospital Stockholm Sweden; ^5^ Applied Biomedical Research Center (CRBA) S. Orsola‐Malpighi Polyclinic Bologna Italy; ^6^ CNR Institute of Molecular Genetics Unit of Bologna Bologna Italy; ^7^ Interdepartmental Center “L. Galvani” (CIG) University of Bologna Bologna Italy

**Keywords:** accelerated aging, anti‐aging strategies, cognitive impairment, Down syndrome, metformin

## Abstract

Down syndrome (DS) has been proposed by George Martin as a segmental progeroid syndrome since 1978. In fact, DS persons suffer from several age‐associated disorders much earlier than euploid persons. Furthermore, a series of recent studies have found that DS persons display elevated levels of age biomarkers, thus supporting the notion that DS is a progeroid trait. Nowadays, due to the progressive advancements in social inclusion processes and medical assistance, DS persons live much longer than in the past; therefore, the early‐onset health problems of these persons are becoming an urgent and largely unmet social and medical burden. In particular, the most important ailment of DS persons is the accelerated cognitive decline that starts when they reach about 40 years of age. This decline can be at least in part counteracted by multi‐systemic approaches including early‐onset cognitive training, physical activity, and psychosocial assistance. However, no pharmacological treatment is approved to counteract this decline. According to the most advanced conceptualization of Geroscience, tackling the molecular mechanisms underpinning the aging process should be a smart/feasible strategy to combat and/or delay the great majority of age‐related diseases, including cognitive decline. We think that a debate is needed urgently on if (and how) this strategy could be integrated in protocols to face DS‐associated dementia and overall unhealthy aging. In particular we propose that, on the basis of data obtained in different clinical settings, metformin is a promising candidate that could be exploited to counteract cognitive decline in DS.

**Key points**

- Down syndrome is the most common genetic cause of intellectual disability and appears to be characterized by an accelerated aging, affecting in particular the central nervous system, with a number of features that overlap with Alzheimer’s disease.
- Social inclusion and cognitive training programs have greatly improved DS cognitive performances over the last decades; however, this advancement is largely frustrated by an early‐onset of an Alzheimer‐like dementia.
- No approved treatment exists so far to counteract such peculiar cognitive condition. A number of clinical trials have been performed but with unsatisfactory results. Based on literature data, we propose that the use of metformin could be worth investigating.

## 1 INTRODUCTION

Down syndrome (DS) (OMIM #190685) is a genetic disorder caused by a partial or complete trisomy of chromosome 21. It is the most common genetic cause of intellectual disability, which is typically associated with well‐defined phenotypic characteristics including distinctive facial features and major congenital malformations.

Life expectancy of DS persons has dramatically increased from 12 years in 1949 to 60 years in 2004, and it is expected to further increase in the near future (Bittles & Glasson, 2004; Glasson et al., 2002). It is worth noting that such extension of the lifespan was of greater amplitude than that occurring in the same years in the general population, suggesting that the extremely short life expectancy of DS persons in the past was not only due to the absence of effective medical treatments but also due to sociocultural factors. In fact, the absence of biological knowledge and social repulsion pushed in the past the medical community to consider the extremely short life expectancy and poor health of these persons as inherent, nonmodifiable traits of the syndrome. On the contrary, when these persons are properly attended, not only does their life expectancy increase spectacularly, but also the alterations at molecular and cellular levels (e.g., immune alterations) result as less dramatic than expected (Franceschi et al., 1978).

The increased life expectancy unfortunately is not paralleled by an increased health span and DS face unhealthy aging characterized by multimorbidity conditions, likely caused by the DS‐accelerated aging phenotype. The progeroid aspects of DS were already postulated by the pioneering work of George Martin in 1978. Today, an indirect but eloquent clue supporting the idea that DS is characterized by an accelerated aging comes from routine clinical practice, as DS persons move directly from pediatrics to geriatrics, skipping almost completely the relatively long period of healthy adulthood present in euploid persons.

At least three different layers of information support the view of DS as a disorder of accelerated aging: (a) clinicopathological features; (b) data showing high levels of markers of biological age in DS; and (c) data showing an acceleration in DS of the molecular mechanisms of aging. In the next sections, we will briefly summarize current knowledge for each of these layers. Starting from this evidence, we will discuss the possible strategies to hinder this accelerated aging, especially for cognitive functions, with a special focus on metformin as a candidate molecule.

## 2 CLINICOPATHOLOGICAL FEATURES OF ACCELERATED AGING IN DS

Accelerated aging in DS is evident in particular at the level of the central nervous system, and by the age of about 50 years, most of DS persons suffer from an early‐onset Alzheimer‐like dementia. Actually, dementia is the second more frequent medical condition after visual impairment present in adult DS persons (Capone et al., 2018), and by far the most relevant health problem, as it entails a loss of independence, a dramatic decrease in the quality of life, and it represents a risk factor of mortality, together with mobility restrictions and epilepsy (Coppus et al., 2008). Typical neuropathological hallmarks of Alzheimer’s disease (AD), including deposition of senile plaques containing amyloid beta‐peptide (Aβ), chronic oxidative stress, and neurofibrillary tangles composed of hyperphosphorylated tau protein are already present by the age of 40 years in DS persons (Lott, 2012). According to a recent study, the diagnosis of dementia is posed on average at 55.8 years (*SD *±6.29) and median survival time after diagnosis is 3.78 years, with some differences between males and females (Sinai et al., 2018). The gene for amyloid precursor protein (APP) is triplicated in DS, being present in chromosome 21. Consequently, DS dementia is believed to be related to the overproduction of APP and consequent deposition of amyloid rather than to a reduced clearance of Aβ, as it occurs in sporadic AD. Therefore, DS dementia is considered more similar to familiar than sporadic forms of AD (SAD). This tenet is also supported by consistent differences between DS and SAD including Aβ deposition patterns, characterized by an increase in deposition of Aβ42 and diffused subcortical plaques in DS (Fukuoka, Fujita, & Ito, 1990; Kida, Wisniewski, & Wisniewski, 1995), and neurological symptoms such as seizures and incontinence, which tend to emerge earlier in DS (Zis & Strydom, 2018). Finally, from a clinical point of view, DS persons appear to be somewhat protected from cerebral amyloid angiopathy and consequent risk of hemorrhage and stroke (Zis & Strydom, 2018). When comparing DS dementia with AD forms due to APP gene microduplication, other differences in the clinical phenotype have been reported (Zis & Strydom, 2018). Despite these important differences between DS dementia and AD, typical signs of cognitive decline and neuropsychological features of dementia (lowered scores of battery tests for language and short memory skills, frontal lobe functions, visuospatial abilities, and adaptive behavior) appear early in DS persons (Arvio & Luostarinen, 2016; Ghezzo et al., 2014).

The immune system is also rapidly deteriorating in DS. It is known that adult DS persons display a series of early‐onset changes that apparently recapitulate in a shorter timescale the normal aging process of the immune system, such as diminished NK cell activity, decreased number of T and B lymphocytes, erosion of telomeres in T lymphocytes, decreased response to mitogenic stimuli of blood leukocytes, and increased risk of autoimmune disorders, among others (Cuadrado & Barrena, 1996; Kusters, Verstegen, & Vries, 2011).

Many other age‐associated diseases display an early onset in DS persons, including thyroid disorders (Prasher, 1999), osteopenia and osteoporosis (Carfì et al., 2017), overweight (Rubin, Rimmer, Chicoine, Braddock, & McGuire, 1998), visual and hearing impairment (Kinnear et al., 2018), epidermal thickening (Kinnear et al., 2018), cardiac atrophy, and moderate diastolic dysfunction (Vis et al., 2012). In many cases, these pathologies occur simultaneously (Capone et al., 2018; Kinnear et al., 2018). A possible exception to this rule is the incidence of solid cancer. Actually, when testis and perhaps other male genital cancers and stomach cancers are excluded, the incidence of solid tumors in adult DS persons is markedly lower with respect to age‐matched controls (Nižetić & Groet, 2012). This finding is paradoxical if we consider that DS has many factors predisposing to cancerogenesis, including chromosome instability, increased DNA damage, and defective DNA repair systems, as well as the presence on chromosome 21 of a number of oncogenes (Nižetić & Groet, 2012). This paradox likely reflects our lack of knowledge regarding anti‐oncogenic mechanisms related to the trisomic condition and will not be further discussed here.

## 3 DS PERSONS ARE CHARACTERIZED BY HIGH LEVELS OF MARKERS FOR BIOLOGICAL AGE

A biomarker of age is defined as a biological parameter (or a combination of biological parameters) able to predict age‐related functional decline and lifespan better than chronological age (Baker & Sprott, 1988; Butler et al., 2004; Johnson, 2006). Different potential biomarkers of age have been proposed so far, and many of them have been shown to grasp age‐acceleration effects (i.e., higher biological age), including cognitive decline and AD (Jylhävä, Pedersen, & Hägg, 2017).

So far, four different biomarkers of age (namely telomere shortening, GlycoAgeTest, Horvath's epigenetic clock, and brain predicted age) have been analyzed in DS persons. As described in the following paragraphs, the results of these independent studies concordantly point for an age‐acceleration effect in DS, indicating that DS persons are biologically older than expected from their chronological age.

Telomere attrition is a well‐established hallmark of aging. Despite some conflicting results (Sanders & Newman, 2013), telomere shortening has been generally associated with age‐related physical/functional decline and with mortality. It has been demonstrated that DS persons have shorter telomeres than age‐matched controls (Vaziri et al., 1993). Subsequent studies showed that telomere attrition is associated with development of dementia in DS persons (Jenkins et al., 2006, 2010), also in longitudinally assessed cohorts (Jenkins et al., 2017), resembling the telomere shortening observed in AD patients (Forero et al., 2016).

The GlycoAgeTest derives from the relative amounts of two plasma *N*‐glycans, measured by means of a DNA sequencer‐aided, fluorophore‐assisted carbohydrate electrophoresis (DSA‐FACE) technique. This biomarker reliably increases with age after 40 years (Dall'Olio et al., 2013; Vanhooren et al., 2009, 2007), and it is likely to be indicative of the biological age of an individual, as patients with Cockayne syndrome and with dementia were shown to have higher GlycoAgeTest values than age‐matched healthy controls (Vanhooren et al., 2010). We recently investigated *N*‐glycomic profiles in 76 DS persons of different ages, as compared with their mothers and siblings (Borelli et al., 2015), and demonstrated that GlycoAgeTest values resulted increased in DS persons as compared to their siblings, in particular at young age. Moreover, GlycoAgeTest values were negatively correlated with Performances IQ score (Borelli et al., 2015).

Epigenetic aging biomarkers, which rely on the widespread DNA methylation changes occurring with age, have proven to be associated with a variety of age‐related conditions, including cognitive decline and AD, and with mortality (Field et al., 2018; Horvath & Raj, 2018). One of the most popular and widely used epigenetic biomarkers of age, Horvath's epigenetic clock (Horvath, 2013), has been applied to four independent sets of specimens from DS persons, including whole blood, total peripheral blood leukocytes (PBL), buccal epithelial cells, and brain postmortem biopsies. According to this study, DS persons resulted as significantly older than their calendar age, with an age acceleration ranging from 2.8 years in buccal cells to 11.5 years in brain (Horvath et al., 2015).

Notably, the same trend of accelerated aging in DS brains has been recently reported by using a totally different approach, that is, magnetic resonance imaging (MRI; Cole et al., 2017). This aging biomarker, based on a machine‐learning guided analysis of MRI data (Cole et al., 2017), was previously associated with mild cognitive impairment and AD (Franke & Gaser, 2012; Gaser, Franke, Klöppel, Koutsouleris, & Sauer, 2013). By this method, Cole et al. observed a brain predicted age difference (brain‐PAD) of 7.69 years between adult DS persons (mean age 42.3 ± 8.7 years) and age‐matched controls (Cole & Franke, 2017). Moreover, in DS persons brain‐PAD correlated with evidence of beta‐amyloid deposition (assessed with Positron Emission Tomography imaging) and with cognitive impairment evaluated as CAMCOG score (Cole et al., 2017).

Serum and urine metabolites can be useful biomarkers of age as well. In a study on serum metabolomics, six serum metabolites were identified as part of a putative signature of aging and measured also in 53 DS persons (average age 28.3 years; Collino et al., 2013). Of these six metabolites, 1‐O‐alkyl‐2‐acylglycerophosphocholine 32:0, sphingomyelins 24:1 and 16:0 did not change in concentration as compared to age‐matched controls, while tryptophan and lysophosphatidylcholines 18:2 and 20:4 had levels closely matching those of elderly and centenarian subjects (Collino et al., 2013).

## 4 MOLECULAR MECHANISMS OF AGING ARE ALTERED IN DS PERSONS: THE SEVEN PILLARS OF AGING

As discussed in the previous paragraph, different biomarkers of age are concordantly altered in DS persons. Moreover, several observations suggest that the main molecular mechanisms involved in the aging process are markedly altered in DS. These mechanisms, also referred as “the seven pillars of aging” (Kennedy et al., 2014), include metabolism, stem cells and regeneration, macromolecular damage, inflammation, adaptation to stress, proteostasis, and epigenetics. DS persons display remarkable alterations for each of these “pillars,” thus supporting the concept of DS as an accelerated aging syndrome (Table 1). However, it is to note that in the majority of these studies, the different pillars were not evaluated in cohorts of different ages, or in longitudinal settings. Therefore, it is difficult to determine whether the alterations observed in DS are inherent to the trisomy rather than a sign of accelerated aging, or whether there is an interaction between the syndrome and the aging process. Further studies are needed to specifically address this issue.

**Table 1:** Alterations of the aging molecular mechanisms (pillars) in DS

Aging pillars	DS phenotype
Metabolism	↑ mTOR pathway activation (Di Domenico et al., 2018) ↑ oxidative stress (Garlet et al.., 2013; Valenti, Manente, Moro, Marra, & Vacca, 2011) ↑ mitochondrial dysfunction (Conte et al., 2018; Helguera et al., 2013) widespread age‐related deregulation of red blood cell metabolism (Culp‐Hill et al., 2013)
Stem cells and regeneration	↓ self‐renewal of stem cells (Adorno et al., 2013; Cairney et al., 2009)
Macromolecular damage	↑ oxidative damage (Cenini et al., 2012; Franceschi et al., 1992) ↑ sensitivity to DNA damaging agents (Morawiec et al., 2008)
Inflammation	↑ chronic inflammation (Zhang et al., 2017) ↑ accumulation of immune cells with memory phenotype (Cossarizza et al., 1990, 1991)
Adaptation to stress	↑ difficulties to cope with increased proteomic stressors (Aivazidis et al., 2017)
Proteostasis	↓ proteostasis (Aivazidis et al., 2017; Di Domenico et al., 2013)
Epigenetics	Age‐related changes in epigenetic machinery (Ciccarone et al., 2018) ↑ DNA methylation age (Horvath et al., 2015)

Taken together, these data support George Martin's pioneering intuition that DS persons suffer from accelerated aging, affecting in particular the nervous system. In the next paragraph, we will discuss how this knowledge could be exploited in the search for new DS therapeutic targets.

## 5 HOW TO FACE THE COGNITIVE DETERIORATION OF DS PERSONS?

Considering that DS persons become dependent on other people's support at a relatively young age because of dementia, and that the parents and families of DS persons also grow old and in some cases cannot take care of them anymore, dementia in adult DS persons is becoming not only an individual clinical problem but also a huge family and public health emergency. It is now clear that integrated interventions including social inclusion processes, schooling, and cognitive training can be of great importance to improve cognitive capabilities of DS persons. In particular, early programs of cognitive training have been demonstrated to counteract effectively cognitive deficits, by improving intellectual skills of DS persons (Connolly, Morgan, Russell, & Fulliton, 1993; Couzens, Haynes, & Cuskelly, 2012).

No pharmacological intervention is at present approved for the amelioration of cognitive deficits of DS (Dierssen, 2012; de la Torre & Dierssen, 2012); however, a number of trials have been performed by using drugs for AD, like acetylcholinesterase inhibitors (donepezil, rivastigmine, galantamine), GABAergic antagonists (pentetrazol), *N*‐methyl‐d‐aspartate receptor antagonists (memantine), or microaliments (minerals, vitamins, antioxidants) that had little or no success (Lott et al., 2011; de la Torre et al., 2016). More recently, another promising molecule has been tested, that is, epigallocatechin gallate (EGCG), a natural polyphenol present in green tea leaves. EGCG is a potent inhibitor of DYRK1A, one of the triplicated genes believed to be involved in the brain alterations observed in DS. EGCG has been tested in vitro and in clinical trials, alone or in combination with cognitive training programs (de la Torre et al., 2016, 2014). However, the beneficial effects of EGCG appear largely inconsistent (Stagni et al., 2017; Stringer, Goodlett, & Roper, 2017) and other studies are granted to confirm the efficacy of this approach. Another possible pharmacological approach is based on inhibitors of GABA_A_ receptors or other modulators of GABA_A_‐mediated transmission, such as NKCC1 (Contestabile, Magara, & Cancedda, 2017). In fact, studies on DS animal models have demonstrated that GABA_A_ antagonists such as pentylenetetrazole (PTZ) are able to improve many cognitive functions of the Ts65Dn mice (novel object recognition, Morris water maze test; Braudeau et al., 2011; Martínez‐Cué et al., 2013), while NKCC1 inhibitors such as bumetanide can improve discriminative memory, spatial and associative memory (Deidda et al., 2015). On these premises, a number of clinical trials have been performed with this kind of drugs. Some of them are still ongoing, and the results are eagerly awaited, while others (NCT02024789 and NCT02484703) showed a good tolerance but a lack of global efficacy (Contestabile et al., 2017).

Despite these somehow disappointing results, the strategy of combining together cognitive training and pharmacological therapies is appealing and certainly deserves attention. However, it is emerging that the rationale used so far (i.e., targeting presumed AD molecular pathways) is not effective, and an alternative approach should be urgently pursued.

We propose that the new vision of Geroscience could be implemented in DS management. According to this approach, aging and age‐related diseases share the same common molecular mechanisms (the seven aging pillars mentioned above), and thus, tackling these mechanisms should in principle counteract both aging and age‐related diseases at a time (Franceschi et al., 2018; Kennedy et al., 2014). Considering that DS persons are likely suffering from an accelerated aging that in turn impinges on the same pillars that are at the basis of physiological aging, as we have discussed thus far, it can be predicted that DS could effectively benefit from the emerging anti‐aging approaches. In this regard, a number of strategies able to impact on the molecular mechanisms involved in the aging process are currently under investigation, including dietary interventions mimicking chronic dietary restriction, drugs that inhibit the growth hormone/IGF‐I axis or the mTOR‐S6K pathway, drugs that activate AMPK or specific sirtuins, and anti‐inflammatory drugs (Longo et al., 2015), some of them being already in clinical trial.

A detailed description of the experimental evidence supporting these strategies has been provided elsewhere (Longo et al., 2015); however, that some of these interventions, in particular regimens of dietary restriction, have been proven to be protective against neurodegenerative pathologies in experimental models. In particular, periodic protein restriction cycles have been shown to promote changes in circulating growth factors and tau phosphorylation associated with protection against age‐related neuropathologies in mice (Parrella et al., 2013). Similarly, calorie restriction has been reported to reduce significantly hippocampal Aβ burden and expression of components of the γ‐secretase complex in female mice of the Tg2576 experimental model of cerebral amyloidosis (Schafer et al., 2015). Finally, many dietary interventions have been shown to ameliorate various cognitive and behavioral tests in rat models of aging and neurodegeneration (Wahl et al., 2017). Accordingly, clinical trials have been performed or are currently ongoing to evaluate the efficacy of such regimens on humans (Horie et al., 2016; Tussing‐Humphreys et al., 2017). However, it can be difficult to obtain sufficient adherence to such dietary protocols in DS persons; therefore, alternative approaches are desirable, including intermittent fasting or calorie restriction mimetics. A prototypical calorie restriction mimetic is rapamycin, a macrolide compound widely used as an immunosuppressant drug. Rapamycin has been demonstrated to extend lifespan in a number of animal models by inhibiting mTOR/S6 pathway. Numerous studies have demonstrated a deregulation of mTOR pathway in DS (Bacalini et al., 2015; Perluigi & Butterfield, 2012) and have linked the alterations of mTOR pathway to cognitive decline, AD, and AD‐like dementia in DS (Di Domenico et al., 2018). There is thus rationale for the use of rapamycin or other rapalogues in conditions where mTOR is altered, including also autistic spectrum disorders and epilepsy (Bockaert & Marin, 2015). However, it has to be considered that rapamycin has important side effects, which can limit its off‐label use as an anti‐aging drug. These include, among others, metabolic dysregulation (e.g., hyperglycemia, hyperinsulinemia, and insulin resistance) and proliferative defects in hematopoietic lineages (Soefje, Karnad, & Brenner, 2011), which could be particularly severe in DS persons.

Another strategy that appears particularly promising is the use of drugs able to selectively kill senescent cells in vivo. Treatments with these “senolytic” drugs are capable of exerting beneficial effects on old animals and delay or block the onset and progression of age‐related diseases (Jeon et al., 2017; Takata et al., 2013). Such an approach could be of great interest for DS persons. A specific investigation on the occurrence of cell senescence in adult DS persons has not yet been conducted; however, it is known that signs of cell senescence are observed in amniocytes and trophoblasts from placentas and embryos with trisomy 21 (Amiel et al., 2013; Biron‐Shental, Liberman, Sharvit, Sukenik‐Halevy, & Amiel, 2015). Moreover, fibroblasts from DS fetuses undergo cell senescence more frequently than fibroblasts from classically developing fetuses, as assessed by senescence‐associated beta‐Gal and p21 expression (Rodríguez‐Sureda, Vilches, Sánchez, Audí, & Domínguez, 2015). However, the strategy with senolytic drugs, though promising, is not yet an option as these drugs are still under preclinical investigation.

Finally, it has been reported that lithium, a drug approved for human use as mood stabilizer, can extend lifespan in animal models through the inhibition of GSK‐3 and activation of NRF2 (Castillo‐Quan et al., 2016); therefore, it could be considered an additional approach to delay the aging process. Interestingly, a number of studies have examined the effects of chronic lithium administration on adult neurogenesis in the Ts65Dn mouse model of DS (Bianchi, Ciani, Contestabile, Guidi, & Bartesaghi, 2010; Contestabile et al., 2013; Faundez et al., 2018; Guidi et al., 2017). These studies indicated that the administration of lithium can restore the neurogenesis in adulthood thus rescuing the synaptic plasticity of newborn neurons, that in turn leads to the recovery of behavioral performance in fear conditioning, object location, and novel object recognition tests (Contestabile et al., 2013), as well as olfactory functions (Guidi et al., 2017). Studies on human cells are lacking; however, we reported years ago that lithium chloride was able to increase the proliferative capability of PBMC from DS subjects after a sub‐optimal stimulation with mitogenic lectins (Licastro et al., 1983). These studies suggest that lithium‐based therapies should be further explored as a potential strategy to counteract cognitive decline of DS persons. At present, we are not aware of human studies or clinical trials with lithium‐based drugs on DS persons, and the numerous possible side effects (including tremors, nausea, vomiting, and feelings of unease) and contraindications (including thyroid disorders, which are very frequent in DS) may discourage this type of approach for DS persons.

## 6 IS METFORMIN A POSSIBLE CANDIDATE FOR COMBATING DS COGNITIVE DECLINE?

Among the molecules that can be considered for their possible anti‐aging effects, there is metformin, a long‐time known biguanide (1,1‐dimethylbiguanide hydrochloride) largely used for Type 2 Diabetes (T2D). Metformin is able to activate AMPK (Takata et al., 2013), a kinase crucial for the regulation of lipid metabolism, cellular glucose uptake, and mitochondria biogenesis, which is believed to mediate the majority of metformin effects on insulin resistance and metabolism. Beside these effects, metformin has also senomorphic activities, that is, the capability to inhibit cell senescence and its related deleterious secretory phenotype (Moiseeva et al., 2013; Noren Hooten et al., 2016). Accordingly, metformin increases lifespan on animal models such as mice (Anisimov et al., 2005, 2011) and *Caenorhabditis elegans* (Cabreiro et al., 2013). A number of data have shown that metformin offers a protection against AD, likely through different mechanisms, including the inhibition of Aβ fibril deposition (Markowicz‐Piasecka et al., 2017), a protein phosphatase 2‐mediated reduction of tau phosphorylation (Kickstein et al., 2010), and a promotion of neurogenesis through the activation of an atypical PKC‐CBP pathway (Wang et al., 2012). Moreover, it is known that AMPK can regulate mTOR, the main inhibitor of autophagy (Ravikumar et al., 2010). Therefore, metformin acts as an anti‐aging drug also by activating autophagy, a process that seems to be deranged in neurodegenerative disorders (Boland et al., 2008; Cataldo, Hamilton, Barnett, Paskevich, & Nixon, 1996). Treatment with metformin is associated with a 51% reduced risk of cognitive impairment (defined by modified Mini‐Mental Status Exam score ≤23) (Ng et al., 2014) and lowers the risk of dementia in T2D patients as compared with other diabetes medications (Cheng et al., 2014; Orkaby, Cho, Cormack, Gagnon, & Driver, 2017). Another study examining the effect of diabetes treatment on specific cognitive domains (verbal learning, working memory, and executive functions) over 4 years showed that only participants who used metformin alone had better cognitive function compared to participants who used other anti‐diabetic drugs (Herath, Cherbuin, & Eramudugolla, 2016). A recent meta‐analysis that considered these and other studies concluded that cognitive impairment is significantly less prevalent in diabetic patients treated with metformin (odds ratio = 0.55, 95% CI 0.38–0.78), and dementia incidence is also significantly reduced (hazard ratio = 0.76, 95% CI 0.39–0.88; Campbell et al., 2018). As a whole, these studies clearly suggest that metformin can effectively counteract neuronal progressive degeneration and dementia; furthermore, it appears that metformin can offer protection also toward other age‐related diseases such as cardiovascular diseases and some types of cancer; therefore, life expectancy of metformin‐treated T2D patients can be higher than age‐matched nondiabetic controls (Amin, Lux, & O'Callaghan, 2018; Bannister et al., 2014; Barzilai, Crandall, Kritchevsky, & Espeland, 2016; Vancura, Bu, Bhagwat, Zeng, & Vancurova, 2018). For these reasons, metformin is going to be tested in the TAME trial on elderly persons with the final goal to evaluate its effects on cardiovascular events, cancer, dementia, and mortality (Barzilai et al., 2016).

In the same vein, and considering its safety profile, metformin could be a promising candidate for possible clinical trials also on DS persons. Preliminary data on the effectiveness of metformin on DS come from a recent in vitro study, where it has been demonstrated that metformin can restore mitochondrial alterations in DS fetal fibroblasts, by inducing the transcriptional coactivator PGC‐1α responsible for mitochondrial biogenesis (Izzo et al., 2017).

DS persons could also benefit from anti‐inflammatory properties of metformin, due to its effects on N‐glycan biomarkers (de Kreutzenberg et al., 2015), on metabolic parameters such as hyperglycemia, insulin resistance, and atherogenic dyslipidemia, but also on the inhibition of NF‐κB activation, ROS, and advanced glycation end‐products (AGEs) formation (Saisho, 2015). Indeed, it has been reported that DS brain displays signs of neuroinflammation (Wilcock et al., 2015), and that DS persons are characterized by chronically elevated levels of some inflammatory markers such as IL‐1β, IL‐6, and TNF‐α since young age (Iulita et al., 2016). Such markers are elevated also in AD patients and correlate with low brain functions. In particular, IL‐6 and C‐reactive protein resulted associated with a decline over time of cerebral function, assessed as blood flow with PET, in regions important for cognition such as the orbitofrontal cortex and hippocampus (Warren et al., 2018). Another astrocyte cytokine, S100B, is elevated in both DS and AD, and it has been reported to induce the synthesis of APP, which can activate microglia that in turn produces IL‐1β (Barger & Harmon, 1997; Li et al., 1998). Thus, excess levels of neural IL‐1β and S100B can influence the neuropathogenesis of AD in DS (Mrak & Griffin, 2004). The progressive, age‐related increase in circulating pro‐inflammatory mediators has been conceptualized as *inflammaging*, and it fuels/triggers many age‐related diseases (Franceschi et al., 2000; Franceschi & Campisi, 2014). It is therefore conceivable that DS persons are characterized by a peculiar form of inflammaging and that the AD‐like dementia occurring in adult DS persons is at least in part sustained by inflammatory cytokines leading to the production and maturation of amyloid plaques.

Finally, it is known that hypothyroidism is one of the most frequent conditions present in adult DS persons, and to this regard, metformin has been reported to decrease the levels of TSH in hypothyroid patients, thus offering a potential protection against enlargement of thyroid gland, goiter, and nodules (Meng, Xu, Chen, Derwahl, & Liu, 2017).

## 7 CONCLUSIONS

Past attempts to face cognitive decline and dementia in DS persons have largely failed. We think it is time for a change in strategy, and, while waiting for specific targets of the AD‐like dementia, we should try to tackle accelerated aging, under the assumption that this approach will also combat related ailments such as dementia. This strategy should possibly include, other than lifestyle and personalized approaches, also dietary interventions and drugs that have proven effective in (or at least good candidate for) delaying/combating the aging process. We have briefly discussed experimental evidence that in particular metformin could be a candidate molecule. In fact, at variance with past approaches, metformin has proven to be effective in preventing a number of phenomena related to both AD dementia and aging at a time. This does not exclude other molecules and therapeutic approaches, and our aim with the present paper was to stimulate an international debate on this topic, in order to obtain a broad consensus finalized to promote appropriately designed rigorous trials, capable of taking into account the clinical heterogeneity of DS persons, particularly when they grow old (Lott, 2012). This scientific debate is also of critical urgency to protect DS persons and their families from a “do‐it‐yourself” approach to this new generation of anti‐aging treatments as well as from underpowered investigations. This debate should involve not only the scientific community, but also families, caregivers, and patient's associations. Owing to the clinical complexity and social implications of DS, we strongly encourage the participation of experts in different fields (pediatricians, neurologists, pharmacologists, biogerontologists, geriatricians, geneticists, and psychiatrists, among others). Considering the complex ethical and clinical issues/caveats related to such trials, pilot proof of principle studies with adequate power should provide guidelines for more extended studies regarding the type of intervention, the age at which it should start, schedule, doses, etc. We are aware that this topic is a delicate issue and that there are open questions still unanswered regarding not only the efficacy of this possible treatment, but also its side effects, permanence after discontinuation, and the cognitive functions that can be actually modified/preserved. Finally, last but not least, we think that the opening of such an international debate would be an opportunity for the scientific community to start discussing the possibility of extending this type of strategy to other types of patients including elderly people with early signs of cognitive impairment.

## CONFLICT OF INTEREST

The authors have nothing to disclose.

## AUTHORS’ CONTRIBUTION

CF and SS involved in concept, analysis of literature, and writing of the manuscript; PG, MGB, NG, and MC performed analysis of literature and critical discussion.
